# Therapist-Guided Telerehabilitation for Adult Cochlear Implant Users: Developmental and Feasibility Study

**DOI:** 10.2196/15843

**Published:** 2020-05-28

**Authors:** Christiane Völter, Christiane Schirmer, Dorothee Hinsen, Marieke Roeber, Stefan Dazert, Kerstin Bilda

**Affiliations:** 1 Department of Otorhinolaryngology, Head and Neck Surgery St. Elisabeth Hospital Ruhr University Bochum Bochum Germany; 2 Kampmann Hearing Aid Acoustics Bochum Germany; 3 Hochschule für Gesundheit University of Applied Sciences Bochum Germany

**Keywords:** telerehabilitation, cochlear implantation, computer-based auditory training, multimodal platform system

## Abstract

**Background:**

Cochlear implants can provide auditory perception to many people with hearing impairment who derive insufficient benefits from hearing aid use. For optimal speech perception with a cochlear implant, postoperative auditory training is necessary to adapt the brain to the new sound transmitted by the implant. Currently, this training is usually conducted via face-to-face sessions in rehabilitation centers. With the aging of society, the prevalence of age-related hearing loss and the number of adults with cochlear implants are expected to increase. Therefore, augmenting face-to-face rehabilitation with alternative forms of auditory training may be highly valuable.

**Objective:**

The purpose of this multidisciplinary study was to evaluate the newly developed internet-based teletherapeutic multimodal system Train2hear, which enables adult cochlear implant users to perform well-structured and therapist-guided hearing rehabilitation sessions on their own.

**Methods:**

The study was conducted in 3 phases: (1) we searched databases from January 2005 to October 2018 for auditory training programs suitable for adult cochlear implant users; (2) we developed a prototype of Train2hear based on speech and language development theories; (3) 18 cochlear implant users (mean age 61, SD 15.4 years) and 10 speech and language therapists (mean age 34, SD 10.9 years) assessed the usability and the feasibility of the prototype. This was achieved via questionnaires, including the System Usability Scale (SUS) and a short version of the intrinsic motivation inventory (KIM) questionnaires.

**Results:**

The key components of the Train2hear training program are an initial analysis according to the International Classification of Functioning, Disability and Health; a range of different hierarchically based exercises; and an automatic and dynamic adaptation of the different tasks according to the cochlear implant user’s progress. In addition to motivational mechanisms (such as supportive feedback), the cochlear implant user and therapist receive feedback in the form of comprehensive statistical analysis. In general, cochlear implant users enjoyed their training as assessed by KIM scores (mean 19, SD 2.9, maximum 21). In terms of usability (scale 0-100), the majority of users rated the Train2hear program as excellent (mean 88, SD 10.5). Age (*P*=.007) and sex (*P*=.01) had a significant impact on the SUS score with regard to usability of the program. The therapists (SUS score mean 93, SD 9.2) provided slightly more positive feedback than the cochlear implant users (mean 85, SD 10.3).

**Conclusions:**

Based on this first evaluation, Train2hear was well accepted by both cochlear implant users and therapists. Computer-based auditory training might be a promising cost-effective option that can provide a highly personalized rehabilitation program suited to individual cochlear implant user characteristics.

## Introduction

Hearing impairment is a major public health problem that affects one third of people aged 65 years or older worldwide, and its prevalence is expected to increase in the future in line with global demographic shifts toward an older population [1,2]. The impact of age-related hearing loss is enormous and extends beyond simply not hearing, as people with impaired hearing have a higher risk for cognitive decline, depression, and frailty [3-5]. Hearing restoration via cochlear implant provision has become a well-accepted treatment option for people of all ages with sensorineural hearing loss, enabling many users to achieve open-set spoken word recognition [6,7].

Typically, a cochlear implant is activated and fit to the recipient 4 weeks after implantation. After cochlear implant activation, (re)habilitation begins, including active auditory training as a vital component [8,9]. This step is necessary because the brain must adapt to the new auditory signal, which differs from the auditory signal that it has been accustomed to [10-12]. In general, there are two different auditory training approaches: (1) the analytic bottom-up approach, which is based on the presentation of paired sounds to train specific skills; and (2) the synthetic top-down approach, which uses sentence identification or text comprehension to improve the cochlear implant users’ overall communication skills [13,14]. The difficulty level of the training has to be built up in a hierarchical manner starting with the simple detection of sound and the discrimination between different signals, progressing to the identification of a sound and understanding complete sentences, even in the presence of background noise [15].

Rehabilitation sessions are conducted in a face-to-face manner in specialized clinical settings [16]. At present, this arrangement works well; however, it may not be as effective or convenient in the future given the finite availability of clinicians/therapists along with the expected increase in the number of cochlear implant users in line with population aging, longer lifespans, and expanding candidacy criteria [17,18]. Further, cochlear implant users may face several potential obstacles in accessing face-to-face rehabilitation sessions, including inadequate reimbursement of the cost-intensive therapeutic sessions by (public) insurance, problems reaching the clinic due to mobility difficulties or geographic distance, and possible comorbidities [19].

Digital media has now become a part of everyday life [20], and electronic health has been one of the fastest growing economic sectors with potential to improve the accessibility of speech and language pathology services [21]. Furthermore, surveys among people with hearing impairment have clearly demonstrated that the majority of subjects are interested in teletherapeutic listening training because it would enable them to train at any place and time [22].

To date, teletherapeutic approaches have mostly focused on patients with neurogenic disorders, and teletherapy was reported to be as effective as standard face-to-face regimes for people with aphasia [23-26]. However, computer-based auditory rehabilitation for people with a cochlear implant is still at an early stage, especially in the German-speaking world [27,28]. By contrast, several English-language, computer-based auditory training programs exist for adults with hearing loss (hearing aid or cochlear implant users), which are primarily intended to supplement, and not to replace, standard face-to-face therapy [29,30].

Most listening programs are self-directed, such as the well-known Listening and Communication Enhancement (LACE; NeuroTone, Redwood City, CA, USA) structured program with interactive and adaptive tools [31-34]. In addition, Speech Perception Assessment and Training System (SPATS; Communication Disorders Technology Inc, Bloomington, IN, USA) is based on a defined training schedule and includes both analytic and synthetic elements [35]. Computer-Assisted Speech Training (CAST) was developed by the Emily Shannon Fu Foundation with free access via Angel Sound (New York, NY, USA), which incorporates a large variety of speech materials and training protocols, along with various mechanisms concerning audio-visual feedback and adaptivity [36].

A critical factor of home-based training is the user’s adherence to the training program [29,37,38]. An important mechanism to encourage people with a chronic illness—who are often driven by external motivation—to persevere with training/rehabilitation is to convert their motivation from external to internal [37]. According to the self-determination theory of Ryan and Deci [39], an individual’s experiences of autonomy, competence, and relatedness are the main elements of motivation. Thus, fostering these experiences is integral to the success of a rehabilitation program [37,39]. Furthermore, the cochlear implant user’s compliance with the program and the usability of the program, which determines the interaction between person and machine, contributes to the variability in training outcome [31]. In short, the user must be self-motivated and the program must be usable and useful for a successful outcome. For this reason, the end users have to be included in the development process to rule out barriers that might hamper the uptake of the new technology by the health care professionals and the patients, whose attitudes and needs may differ [40,41].

In this regard, the purpose of the present multidisciplinary research project was to develop a highly individualized and structured internet-based teletherapeutic system (Train2hear) for auditory rehabilitation and to evaluate the system’s usability and feasibility.

## Methods

### Search Strategy

To identify the existing German-language computer-based auditory training programs for adult cochlear implant users and to guide our development of Train2hear, we searched various scholarly search databases (MEDLINE, Embase, PubMed, Cochrane, Google Scholar). We were particularly interested in the training modalities, delivery system, and theoretical background of published programs.

### Content of the Training

The principle elements of the Train2hear platform were defined according to theoretical auditory rehabilitation concepts, auditory processing models, and the multimodal biopsychosocial concept set forth in the International Classification of Functioning, Disability and Health (ICF) proposed by the World Health Organization [10,42]. A general training schedule was established with different types of exercises that covered various linguistic modalities, which were further split into different tasks. Adaptivity and feedback mechanisms were analyzed and selected. Learning and motivational concepts were evaluated with regard to aural rehabilitation and then adapted to the new training platform.

### User Participation

Keeping the user in mind during program development [43], 18 cochlear implant users and 10 experienced speech and language therapists were involved in the entire development process and in this first feasibility study. Once presenting the entire platform to the users, the participants were asked to judge the program after completing two different exercises without guidance of the researcher. The cochlear implant users (13 women, 5 men; mean age 61 years, range 20-84 years) had bilateral deafness and had been using a cochlear implant for a mean of 2.7 years (range 0.5-8 years). The 10 therapists were all women with a mean age of 34 years.

Four questionnaires were used to assess Train2hear: the Bochumer Questionnaire, System Usability Scale (SUS), Short Scale of Intrinsic Motivation (*Kurzskala der intrinsischen Motivation,* KIM), and Therapists' Questionnaire.

The Bochumer Questionnaire was created specifically for this study to assess user experience with Train2hear. This questionnaire contains 33 questions, which all require a “yes” or “no” answer, that cover the following 5 topics: exercise, feedback, statistical features, overall assessment, and relevance. This questionnaire was completed only by the cochlear implant users. For the exercise topic, the users evaluated two exercises, and therefore completed this section twice; for the other sections, the questions were answered once.

The SUS was used to assess the usability of Train2hear [44]. The SUS includes 10 questions requiring responses on a 5-point Likert scale in which the endpoints are “I strongly disagree” and “I strongly agree”. Five statements were associated with an answer of “I strongly agree” to indicate an overall positive assessment of Train2hear. This scoring method was reversed for the other 5 statements in which “I strongly agree” indicated a negative assessment of Train2hear. An answer of “I strongly agree” was worth 4 points and an answer of “I strongly disagree” was worth 0 points; thus, the higher the score, the more positive the assessment. The total score of the SUS is an absolute number based on the answers of all questions given by the total number of participants. A mean score >68 indicates a high level of usability [45]. Furthermore, for each question, an absolute number and percentage is calculated based on the answers of all participants. The SUS was completed by both the cochlear implant users and therapists.

The KIM was used to assess the cochlear implant users’ intrinsic motivation. This questionnaire is the short form of the Intrinsic Motivation Inventory proposed by Wilde et al [46]. The KIM contains 12 questions, which require responses on a 7-point Likert scale in which 1 means “not at all” and 7 means “very true.” The 12 questions are subdivided into 4 sections: interest/enjoyment, perceived competence, perceived choice, and pressure/stress. The maximum score on each subscale is 21. The first 3 sections are positive (ie, the higher the score, the more positive the assessment), and the last section is negative (ie, the lower the score, the more positive the assessment). The KIM was completed by only the cochlear implant users.

The Therapists Questionnaire was created for this study to assess the therapists’ opinion of the quality of the therapeutic concept and the usability of the new hearing platform. This questionnaire contains 29 questions requiring responses on a 5-point Likert scale in which 0 means “not true” and 4 means “very true.” Only the therapists completed this questionnaire.

### Statistical Analysis

For the SUS and the KIM, inferential statistics were employed to determine if age (Kendall tau) or sex (exact *U* test) of the cochlear implant users significantly affected their scores. For the SUS, the *U* test was used to compare the scores of cochlear implant users with those of the therapists for each question separately and for the total group mean. For the KIM, which was only completed by the cochlear implant users, the analysis was performed for each question separately and for the mean of the total for each of the 4 subgroups. Scores for the Bochumer Questionnaire and the Therapist Questionnaire were summarized descriptively by the mean and SD. *P*<.05 was regarded as a statistically significant difference.

### Ethics

This study was conducted in accordance with the Declaration of Helsinki (from 2018 to 2020) and was approved by the Ethics Committee of Ruhr University of Bochum (18-6423-BR and 18-6423_1-BR).

## Results

### Aural Rehabilitation Programs

Our literature search revealed a limited number of different computer-based auditory training programs available in German for cochlear implant users. Most of these programs are designed as additional training to consolidate the training progress of standard face-to-face therapy, which are all cochlear implant user–driven and self-administered. See Multimedia Appendix 1 for a summary of these programs.

### Development of a New Hearing Platform

#### Background

The features and the key elements of the training program were defined according to the theoretical models of auditory processing and speech understanding proposed by McClelland and Elman [47], Gaskell and Marslen-Wilson [48], Erber [15], and Rönnberg et al [49]. The key elements of the training program are Initial Analysis, Feedback, and Motivation.

**Figure 1:**
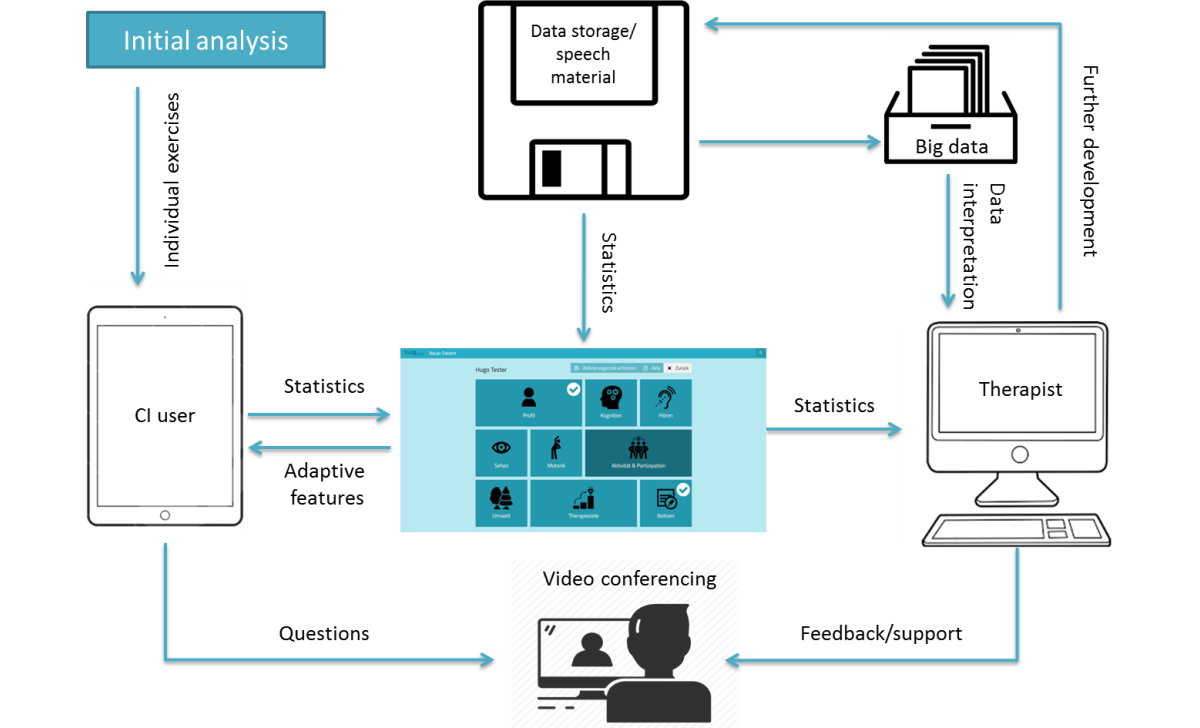
Overview of the concept of the Train2hear hearing platform. CI: cochlear implant.

The basic components of the hearing platform consist of three different interfaces: one for the cochlear implant user, one for the therapist, and one administrative backend that contains all data and speech material (see Figure 1). To enable personal contact between the cochlear implant user and the therapist, a video conferencing feature was included.

#### Initial Analysis

To start the program, the therapist creates an account for the cochlear implant user. The therapist then enters the cochlear implant user’s characteristics into the program (Multimedia Appendix 2). Once this step is completed, a login code is sent to the cochlear implant user.

#### Schedule

**Figure 2:**
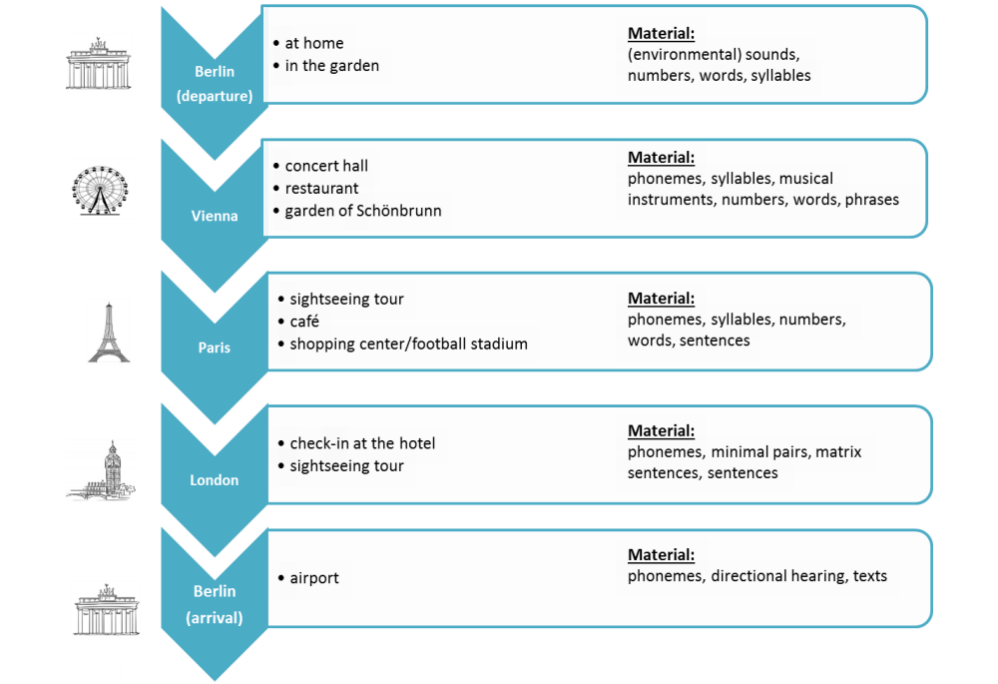
Overview of the training schedule during the Train2hear trip.

**Figure 3:**
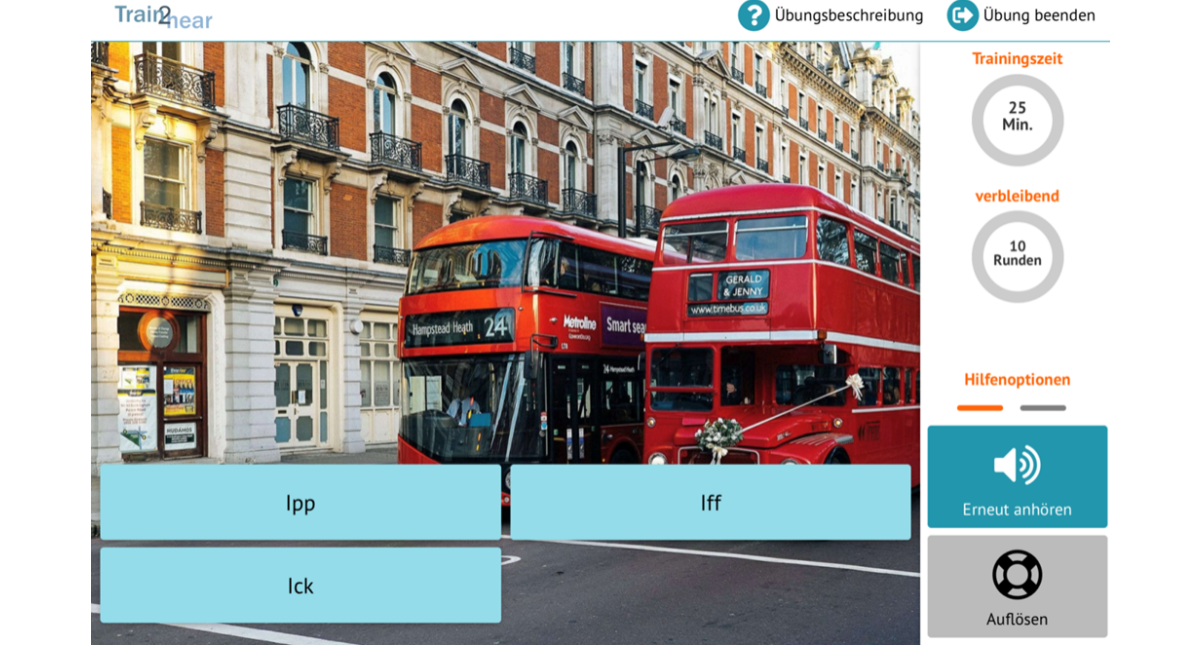
Example of an exercise task (phoneme discrimination).

The training schedule involves a fictitious trip through Europe, which the cochlear implant user follows in a predetermined order (Figure 2). Each city represents a specific auditory level, in which a defined number of exercises must be completed. The scenarios selected at each city are related to everyday life while traveling, such as checking into a hotel, eating in a restaurant, or taking part in a guided tour (Figure 3). During the journey, the cochlear implant user can choose additional exercises such as games, including memory or crossword puzzles.

#### Speech Material

A total of 30 speech tasks were implemented in the training schedule in a hierarchical manner (Figure 2). The speech material used to build up the different tasks covers more than 500 different single words, 600 sentences of different lengths, and about 50 different text messages as well as 500 minimal pairs and 300 syllables spoken by a female and a male speaker. In addition, 50 nonspeech sounds, including musical instruments, were included along with about 25 different background noises with a signal-to-noise ratio ranging from –20 to 20 dB. To prevent learning effects, the audio files are randomly chosen by the program.

#### Adaptivity

**Figure 4:**
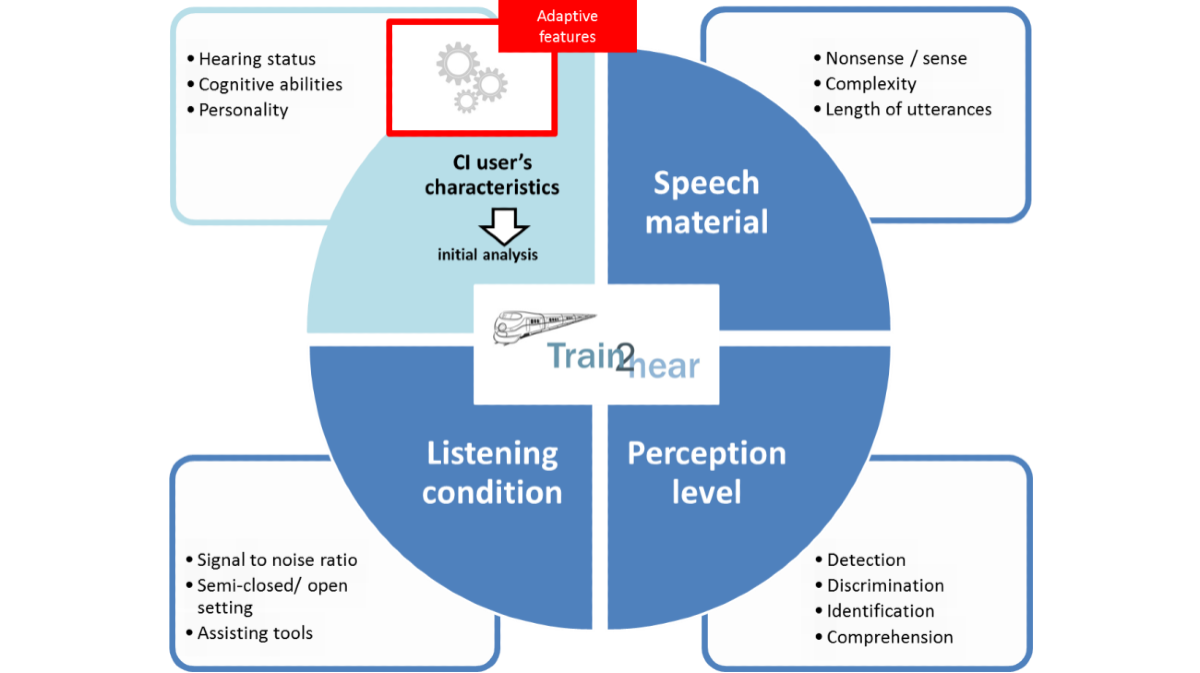
Dimensions of adaptivity (theoretical framework). CI: cochlear implant.

**Figure 5:**
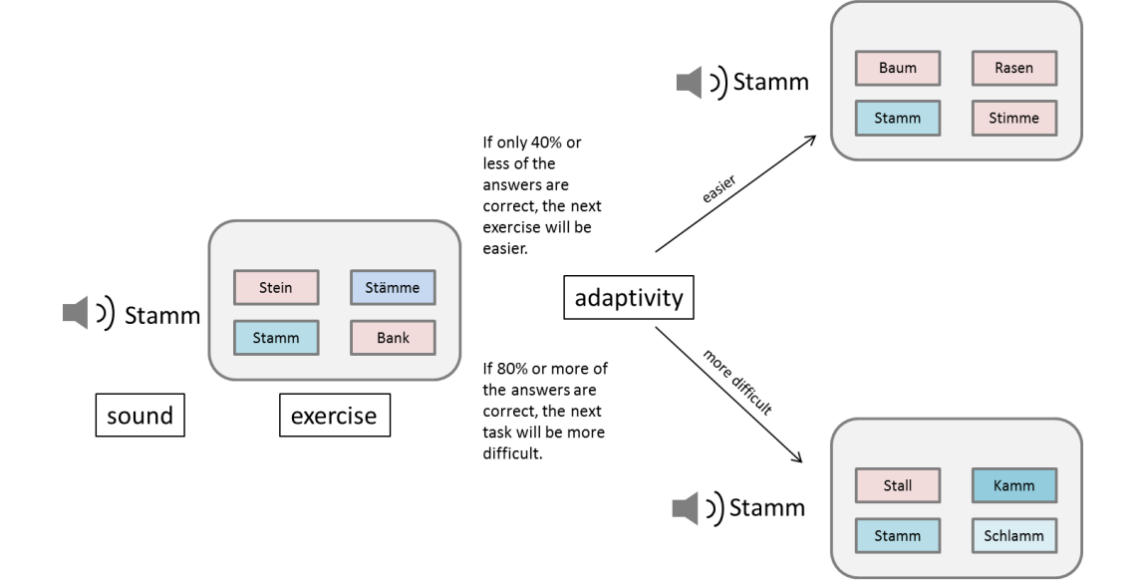
Example of mechanisms of adaptivity.

The cochlear implant users’ metrics such as errors, scores, and task completion times are continually captured during the training. This enables the difficulty of the exercises to be automatically and continually adjusted according to the cochlear implant user’s performance during the exercises. Different mechanisms concerning the speech material, listening conditions, and level of perception have been defined and included for this purpose (Figure 4). Figure 5 presents an example of this adaptivity. When the cochlear implant user’s answers are correct, phonologically similar words are added to make auditory differentiation more challenging; when the performance of the cochlear implant user declines, only words that do not show any phonological similarity are presented.

#### Feedback

Different feedback mechanisms were selected and integrated as supportive elements. Feedback is provided regarding the correctness of the response after completing each exercise. The cochlear implant user’s performance is monitored on a statistics page, which is available to both the user and to the therapist at any time during training. Help functions allow the cochlear implant user to repeat an item up to three times or to suppress the background noise.

#### Motivation

**Figure 6:**
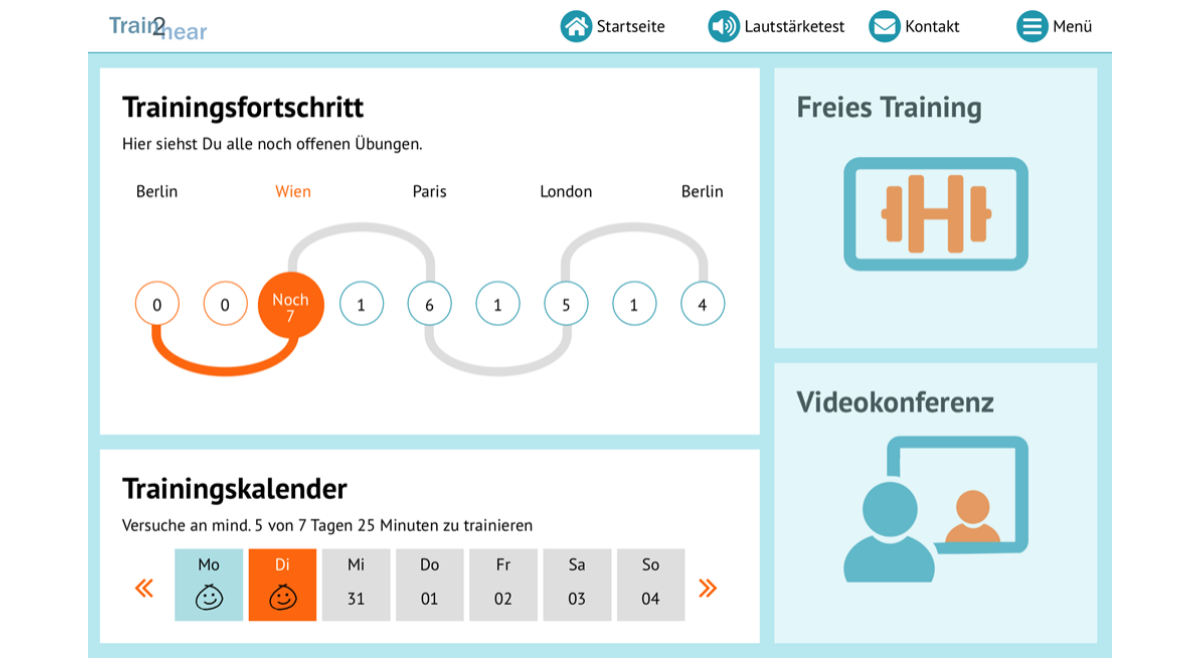
Overview of the cochlear implant user's training schedule.

Motivational enhancement techniques were implemented according to the self-determination theory of Ryan and Deci [39], which is based on competence, autonomy, and relatedness. To promote the feeling of competence, an optimal level of difficulty adapted to the individual patient’s level and positive feedback after each exercise were implemented. Autonomy was encouraged by allowing the user to perform the training anywhere and at any time. The feeling of relatedness was intended to be achieved by specific verbal information and a detailed statistical analysis provided to the cochlear implant user. Furthermore, a train conductor who serves as an avatar along with a calendar about the time spent in the training were added to the program as additional motivational elements (Figure 6).

#### Technical Requirements

Train2hear is a web-based platform designed for a tablet with the mobile operating system iOS and requires access to a wireless network. Passwords are saved in a hashed manner and are indiscernible to outsiders. All data regarding the training are saved for each account separately.

### Questionnaires

#### Bochumer Questionnaire

Overall, the cochlear implant users found that the program met their expectations, that they would recommend it to others, and that they would like to continue using it (items 27-29). Additionally, they reported that the exercises were interesting and relevant to their daily lives (items 30-31).

Multimedia Appendix 3 lists the responses to each item in the questionnaire. A minority of cochlear implant users reported that they had difficulty finding the exercise (item 1) and that they would need more detailed information on their mistakes in order to improve (item 12). Otherwise, the responses were overwhelmingly positive: over 90% of cochlear implant users reported that the exercise was clear (item 2), the function of each button was clear (item 5), images were clear and appealing (items 8 and 9), and feedback was understandable, visually appealing, helpful, motivating, and sufficient (items 11, 13-17). More than 80% of users found the statistical features helpful and clear (items 18-21). A few responses were missing as some cochlear implant users were unsure about the best answer.

#### SUS

The mean total SUS score was 85.3 (SD 10.32) for cochlear implant users and 93.0 (SD 9.17) for therapists. These scores (and the scores for each question) indicate that both groups found that Train2hear has excellent usability (Multimedia Appendix 4) [45].

No significant difference in total mean SUS scores was found according to group (*P*=.05). Therapists had significantly higher mean scores on items 7 (*P=*.04) and 10 (*P=*.02).

Regarding a possible influence of age on usability among cochlear implant users, increased age negatively correlated with SUS score with respect to the total mean percent (*P=*.008) and on items 2 (*P=*.03), 5 (*P=*.009), 7 (*P=*.007), and 10 (*P=*.03). Regarding a possible influence of sex on usability amongst cochlear implant users, men had significantly higher scores than women on item 7 (*P=*.01). Some answers were missing because some cochlear implant users and one therapist were unsure about the right answer. See Multimedia Appendix 4 for the full SUS questionnaire items and associated answers.

#### Intrinsic Motivation

Scores on the interest/enjoyment subsection of the KIM indicated that cochlear impact users found the program interesting and enjoyable. Scores of the pressure/stress subsection (in which, unlike the other subsections, low scores indicate positive feedback) showed that the cochlear implant users did not feel to be under a great deal of pressure while working on the program (Table 1).

Regarding a possible influence of age, increased age negatively correlated with scores on items 7 (*P=*.004), 10 (*P=*.007), 11 (*P=*.02), and 12 (*P=*.01), and on the total score for the pressure/stress subsection (*P<*.001). No significant differences were found according to sex. See Table 1 for the full KIM questionnaire and answers. One user did not answer all questions.

**Table 1:** Intrinsic motivation (KIM^a^) scores for cochlear implant users (N=18).

Item		n (%)	Mean (SD)
**Interest/enjoyment**			
	1. I enjoyed working with the program.	18 (100)	6.50 (0.70)
	2. I found working with the program was very interesting.	18 (100)	6.56 (0.71)
	3. Working with the program was enjoyable.	18 (100)	6.22 (1.06)
	Total	18 (100)	19.28 (2.27)
**Perceived competence**			
	4. I am satisfied with my performance with the program.	18 (100)	5.78 (1.35)
	5. I was skillful when working with the program.	18 (100)	5.67 (1.33)
	6. I think I was pretty good at using this program.	18 (100)	5.50 (1.38)
	Total	18 (100)	16.94 (2.92)
**Perceived choice**			
	7. I was able to manipulate the program myself.	18 (100)	5.22 (1.48)
	8. I could choose how to use the program.	17 (94)	4.77 (2.11)
	9. I could proceed the way I wanted in the program.	17 (94)	4.77 (2.05)
	Total	17 (94)	14.71 (4.87)
**Pressure/stress**			
	10. I felt under pressure while working with the program.	18 (100)	1.39 (1.42)
	11. I felt stressed while working with the program.	18 (100)	2.50 (2.01)
	12. I was not sure if I could work well with the program.	18 (100)	2.33 (1.94)
	Total	18 (100)	6.22 (3.95)

^a^KIM: Kurzskala intrinsischer Motivation; scores are based on a Likert scale in which higher scores indicate more positive answers except for the subsection Pressure/Stress, in which lower scores indicate more positive answers.

#### Therapist Questionnaire

The therapists found that the program was easy to navigate (items 1-2, 15); had exercises that were clear, relevant, and appealing (items 6-11); and provided feedback that was appealing and motivating for cochlear implant users (items 12-14). Overall, for most therapists, Train2hear met their expectations and they could imagine using it in their therapeutic regimes and recommending it to cochlear implant users (items 22-24). Therapists clearly indicated that the program could enhance regular (face-to-face) training but could not replace it (items 25-27). All therapists thought the program was scientifically sound (item 29).

Although the therapists expressed concern over the clarity of the statistics (items 5, 17), they also indicated that the statistical analysis made it easy for cochlear implant users to understand their own performance (item 18). Some answers were missing as one therapist was unsure of the best answer. See Multimedia Appendix 5 for the full questionnaire items and answers.

## Discussion

### Principal Findings

A teletherapeutic computer-based training platform named Train2hear was developed, and its feasibility was primarily assessed with cochlear implant users and therapists. This platform allows adult cochlear implant users to train auditory skills on their own.

New therapeutic concepts such as teletherapy have to be considered to meet the growing demand of speech therapy in the future, especially as a method for augmenting standard face-to-face therapy [50]. The advent of helpful and easy-to-use platforms that cochlear implant users can engage with wherever and whenever they like could save therapists’ time while also empowering cochlear implant users (and their caregivers) by making it easier for them to participate in and benefit from the rehabilitation process [51]. This concept is in line with Mogler et al [52] who recognized the need to involve cochlear implant users, especially those with a chronic condition or disease, in the rehabilitation process.

Train2hear is a well-structured, therapist-guided program. It combines a standardized protocol and a highly individualized schedule that is tailored to the specific demands of cochlear implant users according to the principle of rehabilitation set forth by the ICF [42].

To promote optimal learning, the exercises are intended to be set at an appropriate level of difficulty; that is, sufficiently challenging to maintain motivation but not so challenging that the cochlear implant user becomes discouraged or frustrated [53]. Computer-assisted programs are an ideal option to permanently and automatically adapt to the user’s level during the rehabilitation process [18]. An initial test followed by various mechanisms of adaptivity are therefore core elements of this new auditory training.

As stated by Henshaw et al [37] who analyzed a computerized phoneme discrimination training for individuals with hearing impairment, intrinsic motivation is a key factor with regard to adherence. Thereby, motivational principles to enhance intrinsic motivation have been fully considered in the new training platform [39]. In addition, strong therapeutic guidance is provided to the user by implementing strict instructions, an external control via daily log-in, a videoconferencing tool, and immediate feedback regarding progress during training, as suggested by Humes et al [54].

The results from the questionnaires revealed that both cochlear implant users and therapists viewed Train2hear positively. The cochlear implant users found the training program easy and enjoyable to use, would like to continue using it frequently, and would recommend it to others. Compared to younger users, older users rated usability slightly worse, although their scores still indicated a high level of usability. Older users were also less confident in using the program overall, and claimed to need more technical support. This is in line with previous reports indicating that age is an important variable for computer usage and that gender differences increase with age [55].

Clear introductory videos and technical support via mail, phone, or personal contact should be provided to help older adults cope with a new technology [56]. Moreover, teletherapeutic programs should be tailored to the specific physical or mental barriers faced by older people, such as diminished eyesight or deteriorated motor skills, and factors influencing the acceptance of technology by seniors have to be taken into account in the design [57].

### Comparison to Previous Studies

Most computer-based modules that train auditory functions are offered as mobile apps or web-based training options, which are applied as an adjunct modality to consolidate the training progress. The majority of auditory training programs are not therapeutically guided, with the user instead selecting the type and number of exercises they wish to perform [58]. However, learners have been shown to benefit from a well-structured training program that they follow in a defined order [59]. Thus, the Train2hear program clearly defines the type of tasks the user has to perform and the user can only choose the order the tasks are performed within a given level.

Although some programs do include different levels of difficulty that the user can choose from, none of the available programs automatically adapts to the user’s performance or includes a comprehensive initial evaluation of the user’s strengths and weaknesses. Experts' supervision can only be obtained via email, during an in-clinic visit, or by phone. We implemented a video conference element into the new platform, which enables the therapist to perform a simple consultation and to deliver therapeutic sessions.

### Limitations

A limiting factor of the present work is the small number of therapists and cochlear implant users included in this first evaluation. Another critical point to mention is the user’s adherence to the training [29,37,38]. Interactional and relational processes, which have a great impact on treatment adherence and efficacy of traditional health care, are changed through human-computer interfaces. Given the importance of the user’s attitude to telerehabilitation and the availability of a supporting person to the outcome of training [60], professional and nonprofessional users should both be involved in the developmental process as early as possible to increase the acceptance of telepractice. Initial reluctance is not necessarily an obstacle; indeed, Hines et al [61] demonstrated that mixed feelings of therapists toward telepractice might later change to positive awareness [61].

The cochlear implant users themselves were involved from the beginning of the study via an online survey of their needs and expectations. Furthermore, different mechanisms were implemented to encourage cochlear implant users to adhere to the new platform for long-term training. However, evaluation of adherence was not the primary target in the study design.

### Outlook

In a future study, we will examine the levels of adherence to Train2hear and its effectiveness as a rehabilitation tool, including more participants for a longer period of evaluation.

Computer-based therapy platforms can record a cochlear implant user’s progress in great detail. This external evidence could lead to the creation of better therapeutic interventions and training protocols [17]. Currently, standard face-to-face therapy is mainly based on internal evidence and is highly individualized owing to therapist involvement.

To reduce the time-consuming development of new tasks, future research should focus on automatic creation of items using artificial intelligence.

### Conclusions

Teletherapeutic hearing rehabilitation software such as the new Train2hear platform offers a great opportunity for cochlear implant users and therapists. Although there are still several limitations to overcome and various questions to be answered, this preliminary assessment demonstrates that a standardized but highly individualized computer-based auditory training program might have a great and positive impact on hearing rehabilitation in the near future.

## Appendix

Multimedia Appendix 1 Overview of German computer-based auditory training programs available for cochlear implant users.

Multimedia Appendix 2 Components of the initial analysis based on the International Classification of Functioning, Disability, and Health.

Multimedia Appendix 3 Bochumer questionnaire for cochlear implant users.

Multimedia Appendix 4 System Usability Scale scores for cochlear implant users (N=18) and therapists (N=10).

Multimedia Appendix 5 Results for the Therapist Questionnaire (N=10).

## Abbreviations

CAST: Computer-Assisted Speech Training
ICF: International Classification auf Functioning Disability and Health
KIM: Kurzskala intrinsischer Motivation
LACE: Listening and Communication Enhancement
SPATS: Speech Perception Assessment and Training System
SUS: System Usability Scale
