# Supplementary material for: Therapist-Guided Telerehabilitation for Adult Cochlear Implant Users: Developmental and Feasibility Study
Source: JMIR Rehabil Assist Technol. 2020 May 28;7(1):e15843. doi: 10.2196/15843 (PMC7290457; doi:10.2196/15843)
Supplement: Multimedia Appendix 1 [file rehab_v7i1e15843_app1.pdf]

Overview of German computer-based auditory training programs available for cochlear implant users.

	Hörtraining online Sensoton	Online Hörtraining Verheyen	Asklepios (HCIZ)	Listen Up! (MED-EL)	Sound Success (Advanced Bionics)	OLCIT (Jade University)	Hörtraining DHZ (DHZ)	Schallquelle (Biedermann Gbr)
Synthetic approach	Y[a]	Y	Y	Y	Y	Y	Y	Y
Analytic approach	Y	N[b]	Y	Y	N	N	Y	Y
Structured program	Y	Y	N	N	N	N	N	N
Initial analysis	n.s.[c]	N	N	(Y)[d]	N	N	N	N
Adaptive features	N	N	N	N	N	N	N	N
Therapist guided	N	N	N	N	N	N	N	N
Simple statistical evaluation	Y	N	Y	Y	Y	N	Y	Y
Complex statistical evaluation	n.s.	N	N	N	N	N	N	N

[a]Y: included

[b]N: not included.

[c]n.s: not specified.

[d](Y): Listen up! contains an initial test, based on hearing status and without impact on training schedule.
