# Supplementary material for: Therapist-Guided Telerehabilitation for Adult Cochlear Implant Users: Developmental and Feasibility Study
Source: JMIR Rehabil Assist Technol. 2020 May 28;7(1):e15843. doi: 10.2196/15843 (PMC7290457; doi:10.2196/15843)
Supplement: Multimedia Appendix 2 [file rehab_v7i1e15843_app2.pdf]

Components of the initial analysis based on the International Classification of Functioning, Disability, and Health.

Component	Description
Cochlear implant user profile	Name, address, date of birth, sex, family status, professional activity
Cognition	Working memory, short- and long-term memory, processing speed, attention measured with a cognition test battery
Hearing	Information on hearing loss in both ears, speech recognition measured with the Freiburger numeric and monosyllabic comprehension test, sentence recognition measured with the Oldenburger sentence comprehension test (Hörtech GmbH, Oldenburg, Germany)
Vision	Information on vision status
Motor function	Information on motor functions
Activity and participation	Communication skills, difficulties in conversations, speech comprehension ability on the phone, sound localization, activity restriction due to hearing loss
Environment	Noise environment in everyday life, support from a trusted person, acceptance of wearing/using a cochlear implant
Therapeutic goals	Subdivided into the following areas: speech comprehension, speech perception and identification, sound localization, and quality of life
Notes	Free text session
