# Supplementary material for: Therapist-Guided Telerehabilitation for Adult Cochlear Implant Users: Developmental and Feasibility Study
Source: JMIR Rehabil Assist Technol. 2020 May 28;7(1):e15843. doi: 10.2196/15843 (PMC7290457; doi:10.2196/15843)
Supplement: Multimedia Appendix 3 [file rehab_v7i1e15843_app3.pdf]

Bochumer questionnaire for cochlear implant users.

Question		Total respondents, n (%)	Yes, n (%)	No, n (%)
**Exercises (N=36)**[a]				
	1. Did you have difficulties finding the exercise?	36 (100)	8 (22)	28 (78)
	2. Was the exercise clear?	35 (97)	33 (94)	2 (6)
	3. Did you find the "Audio Repeat" button?	35 (97)	33 (94)	2 (6)
	4. Did you feel supported by the "Audio Repeat" button?	36 (100)	35 (97)	1 (3)
	5. Was the function of each button clear to you?	36 (100)	33 (92)	3 (8)
	6. Did you miss supporting tools to be able to complete the exercise satisfactorily?	36 (100)	3 (8)	33 (92)
	7. Do you think there were enough options to answer?	35 (97)	34 (97)	1 (3)
	8. Did you find the images appealing?	33 (92)	32 (97)	1 (3)
	9. Was every image clear/unambiguous to you?	33 (92)	31 (94)	2 (6)
	10. Did you have the feeling that the level of difficulty of the exercise was changing?	34 (94)	9 (26)	25 (74)
	11. Was the visualization of the feedback comprehensible?	35 (97)	35 (100)	0 (0)
	12. Would you need more detailed information about your mistakes to be able to improve?	34 (94)	7 (21)	27 (79)
**Feedback (N=18)**				
	13. Was the feedback presented in a visually appealing way?	18 (100)	18 (100)	0 (0)
	14. Was the feedback clear to you?	18 (100)	15 (83)	3 (17)
	15. Does the feedback help you to judge your performance?	18 (100)	18 (100)	0 (0)
	16. Does the feedback motivate you to keep on training?	18 (100)	18 (100)	0 (0)
	17. Was there sufficient feedback?	18 (100)	16 (89)	2 (11)
**Statistical features (N=18)**				
	18. Did you like the design of the statistics page?	18 (100)	17 (94)	1 (6)
	19. Is the summarizing table clear?	16 (89)	14 (88)	2 (12)
	20. Do the statistical data help you to assess your performance?	18 (100)	15 (83)	3 (17)
	21. Do the statistical data motivate you to keep on training?	18 (100)	15 (83)	3 (17)

**Overall assessment (N=18)**				
	22. Was the program challenging?	18 (100)	12 (67)	6 (33)
	23. Was the program varied?	18 (100)	18 (100)	0 (0)
	24. Did the program require you to make unnecessary mouse clicks?	18 (100)	0 (0)	18 (100)
	25. Does the program contain unclear terms, names, abbreviations, or symbols?	18 (100)	1 (6)	17 (94)
	26. Is every function/feature clear and understandable?	18 (100)	16 (89)	2 (11)
	27. Did the program meet your expectations?	16 (89)	13 (81)	3 (19)
	28. Would you recommend the program to others?	18 (100)	17 (94)	1 (6)
	29. Would you like to continue to use the program in the future?	16 (89)	14 (88)	2 (12)
**Relevance (N=18)**				
	30. Do you think the exercises are interesting for your daily life?	18 (100)	17 (94)	1 (6)
	31. Do you think the exercises are relevant to you daily life?	18 (100)	15 (83)	3 (17)
	32. Do the exercises differ from other computer based hearing training programs?	13 (72)	8 (62)	5 (38)
	33. Do the exercises differ from the exercises that you perform during the face-to-face therapy (rehabilitation) in the clinic/cochlear implant center?	17 (94)	9 (53)	8 (47)

[a]Most cochlear implant users evaluated the “exercises” twice and the other topics once.
