# Supplementary material for: Therapist-Guided Telerehabilitation for Adult Cochlear Implant Users: Developmental and Feasibility Study
Source: JMIR Rehabil Assist Technol. 2020 May 28;7(1):e15843. doi: 10.2196/15843 (PMC7290457; doi:10.2196/15843)
Supplement: Multimedia Appendix 4 [file rehab_v7i1e15843_app4.pdf]

System Usability Scale scores for cochlear implant users (N=18) and therapists (N=10).

Statement		Respondents, n (%)	0	1	2	3	4	Total, n (%, SD)	Total maximum	P-Value
**1. I think that I would like to use this program frequently**										
	CI[c] users	17 (94)	0	0	2	4	11	60 (88.2, 0.72)	68	.05
	Therapists	9 (90)	0	0	0	0	9	36 (100, 0.00)	36	
**2. I thought the program was easy to use**										
	CI users	17 (94)	0	1	2	4	10	57 (83.8, 0.91)	68	.06
	Therapists	9 (90)	0	0	0	2	7	34 (94.4, 0.44)	36	
**3. I found the various functions in this program were well integrated**										
	CI users	18 (100)	0	0	0	6	12	62 (86.1, 0.49)	72	.28
	Therapists	9 (90)	0	0	1	5	3	29 (80.6, 0.71)	36	
**4. I would imagine that most people would learn to use this program very quickly**										
	CI users	18 (100)	0	0	2	5	11	59 (81.9, 0.71)	72	.04
	Therapists	9 (90)	0	0	0	5	4	31 (86.1, 0.53)	36	
**5. I felt very confident using the program**										
	CI users	18 (100)	0	2	2	5	9	53 (73.6, 1.04)	72	.22
	Therapists	9 (90)	0	0	0	1	8	34 (94.4, 0.44)	36	
**6. I found the program unnecessarily complex**										
	CI users	17 (94)	0	1	0	3	4	62 (91.2, 0.79)	68	.77
	Therapists	9 (90)	0	0	1	3	13	32, (88.9, 0.73)	36	
**7. I think that I would need the support of a technical person to be able to use this program**										
	CI users	18 (100)	3	2	1	3	9	49, (68.1, 1.60)	72	.65
	Therapists	9 (90)	0	0	0	1	8	35 (97.2, 0.33)	36	
**8. I thought there was too much inconsistency in this program**										
	CI users	18 (100)	1	0	0	2	15	62 (86.1, 0.97)	72	.71
	Therapists	9 (90)	0	1	0	1	7	32 (88.9, 1.01)	36	

**9. I found the program very cumbersome to use**										
	CI users	18 (100)	1	0	0	2	18	62 (86.1, 0.97)	72	.13
	Therapists	9 (90)	0	0	0	1	8	35, (97.2, 0.33)	36	
**10. I needed to learn a lot before using the program**										
	CI users	18 (100)	1	3	0	4	10	54 (75.0, 1.11)	72	.02
	Therapists	9 (90)	0	0	0	0	9	36 (100, 0.00)	36	

[a]For statements 1-5, scores range from 0 “I strongly disagree” to 4 “I strongly agree”, whereas the scores are reversed for statements 6-10. In the original questionnaire the statements were ordered 1, 6, 2, 7, 3, 8, 4, 9, 5, 10. For ease of interpretation, we have reordered the statements in the table.
[b]Scores were compared based on the percentage of the total score to the maximum score possible.
[c]CI: cochlear implant.
