# Supplementary material for: Therapist-Guided Telerehabilitation for Adult Cochlear Implant Users: Developmental and Feasibility Study
Source: JMIR Rehabil Assist Technol. 2020 May 28;7(1):e15843. doi: 10.2196/15843 (PMC7290457; doi:10.2196/15843)
Supplement: Multimedia Appendix 5 [file rehab_v7i1e15843_app5.pdf]

Results for the Therapist Questionnaire (N=10)

Please indicate how true each of the following statements is for you			n (%)	Not true 0	1	2	3	Very true 4
**Backend for therapists**								
	**Initial analysis**							
		1. I can easily find my way around the section "My patients".	10 (100)	0	0	0	0	10
		2. I can easily create new accounts for my patients.	10 (100)	0	0	0	0	10
		3. All elements of the initial analysis are covered.	10 (100)	0	0	1	1	8
	**Statistical features**							
		4. The statistics are understandable.	10 (100)	0	0	2	6	2
		5. The statistics are clearly represented.	10 (100)	0	1	4	4	1
**Training program**								
	**Exercises**							
		6. The exercises are relevant to cochlear implant users' daily lives.	10 (100)	0	0	0	4	6
		7. The exercises are relevant to the hearing rehabilitation process.	10 (100)	0	0	0	0	10
		8. The exercises cover the most important elements of auditory training.	10 (100)	0	0	0	3	7
		9. The exercises are clear.	10 (100)	0	0	0	1	9
		10. The supporting tools meet the needs of cochlear implant users.	9 (90)	0	0	1	1	7
		11. The concept of travelling through Europe is appealing.	10 (100)	0	0	0	3	7
	**Feedback**							
		12. The feedback is appropriate for cochlear implant patients.	10 (100)	0	0	0	1	9
		13. With the given feedback, the patients can assess their performance realistically.	9 (90)	0	0	0	3	6
		14. The given feedback seems to be motivating for further training.	9 (90)	0	0	0	3	6
	**Adaptive features**							
		15. Adapting the level of difficulty throughout an exercise is reasonable from a therapist's point of view.	9 (90)	0	0	0	0	9
	**Statistical features**							
		16. The statistical analysis makes it easy for the patients to assess their training performance.	10 (100)	0	0	1	2	7
		17. From the patient's point of view, the statistics are clear and comprehensible.	10 (100)	0	0	3	4	3
	**Motivational features**							
		18. The statistical analysis makes it easy for the patients to assess their training performance.	10 (100)	0	0	1	2	7
		19. The features of the program (training agenda, time information) motivate patients to practice regularly.	10 (100)	0	0	1	6	3

	**Design**							
		20. The digital training program is visually appealing.	9 (90)	0	0	0	2	7
		21. The font size is appropriate.	10 (100)	0	0	0	0	10
**Overall assessment**								
		22. The program met my expectations.	9 (90)	0	2	0	3	4
		23. I could imagine integrating the program in my therapeutic routine.	9 (90)	0	0	1	8	0
		24. I would recommend this program to my patients.	10 (100)	0	0	0	0	10
		25. Patients would be as satisfied with this type of therapy as they are with face-to-face therapy.	9 (90)	0	5	1	3	0
		26. Digital training could add to regular auditory training.	10 (100)	1	0	0	0	9
		27. Digital training could replace regular auditory training.	10 (100)	4	4	2	0	0
		28. Family members would likely support the patients during the digital training.	10 (100)	1	1	1	3	4
		29. The program is scientifically sound.	10 (100)	0	0	0	2	8
